# Noonan syndrome: rhGH treatment and *PTPN11* mutation

**DOI:** 10.1002/mgg3.2266

**Published:** 2023-08-01

**Authors:** Xian Wu, Jiali Wu, Yi Yuan, Li Yang, Lirong Yu

**Affiliations:** ^1^ Department of Endocrinology, Genetics and Metabolism Jiangxi Provincial Children's Hospital Nanchang China; ^2^ Department of Emergency Jiangxi Provincial Children's Hospital Nanchang China

**Keywords:** children, Noonan syndrome, *PTPN11* mutation, recombinant human growth hormone

## Abstract

**Objective:**

To analyze the clinical data and genetic characteristics of Noonan syndrome, both the effect and side effects of recombinant human growth hormone (rhGH) treatment.

**Methods:**

We collected clinical data from 8 children with Noonan syndrome diagnosed from November 2017 to June 2021. The diagnosis was clarified by exome second‐generation sequencing and parental PCR‐NGS validation and interpretation of the preceding evidence, and growth hormone therapy was administered. Of the cases, four males and four females were seen for slow height growth and the median age at diagnosis was 8 years 7 months (1 year 7 months to 12 years 6 months).

**Results:**

Here, 7 children were treated with rhGH. Compared to the pre‐treatment period, the growth rate increased after rhGH treatment [3.7 ± 0.5 cm/year before treatment and 8.0 ± 1.0 cm/year after treatment, *p* < 0.01], with the maximum growth rate between 3 and 6 months of treatment and decreasing with the duration of treatment thereafter. The growth hormone treatment was discontinued and the orthopedic consultation was ordered with regular follow‐up, which was considered to be related to the *PTPN11* mutation.

**Conclusion:**

Noonan syndrome is characterized by slow growth, short stature, mental retardation, peculiar facial features, structural heart abnormalities and abnormal bone metabolism. and osteochondroma was found after case 2 rhGH treatment. Genetic examination is mostly caused by *PTPN11* mutation. It is recommended to pay attention to bone metabolism abnormalities before growth hormone treatment, especially in children with *PTPN11* mutations.

## Abstract 2

We analyze the clinical data and genetic characteristics of Noonan syndrome, both the effect and side effects of recombinant human growth hormone (rhGH) treatment.

## 1 INTRODUCTION

Noonan syndrome is a rare congenital malformation syndrome occurs due to the upregulation of the RAS‐mitogen‐activated‐protein kinase (RAS‐MAPK) signaling pathway (Johnston et al., 2018). This group of disorders is mostly autosomal dominant, but can rarely be autosomal recessive. It has an incidence rate of (4–10) / 10,000 live births (Yart & Edouard, 2018), and is often associated with abnormalities in various bodily systems, including the cardiac, skeletal, urinary, digestive and endocrine systems. Delayed puberty may also be observed in some children. Mutations in 16 genes, including *PTPN11* (50%), *SOS1* (20%) and *RAF1* (5%–15%), have been linked to the development of Noonan syndrome (Kessler et al., 2021; Pagnamenta et al., 2019; Tartaglia et al., 2011; Wong et al., 2023). As the RAS‐MAPK pathway is present in most cells, it regulates important cellular processes like growth factors and hormone secretion, making it a key factor in the syndrome development (Tafazoli et al., 2017).

Research has indicated that adult lifetime heights of individuals with Noonan syndrome who are classified as short are typically below the 3rd percentile or 2 standard deviations from the mean height (Cheung et al., 2017; Kim et al., 2022). There is some observational evidence from abroad that rhGH treatment increases annual growth rates and increases height Standard Deviation Score (SDS) in children with Noonan syndrome short stature during treatment (Şıklar et al., 2016). rhGH is recommended for the treatment of children with Noonan syndrome in combination with short stature (Libraro et al., 2021; Stagi et al., 2022).

In this study, we conducted a retrospective analysis of clinical data from eight children with Noonan syndrome who were admitted in recent years, as well as assessed the effectiveness, treatment regimens and challenges encountered during the process of rhGH treatment. The aim of our study was to provide a useful reference point for clinicians in regards to the diagnosis and management of this disorder.

## 2 SUBJECTS AND METHODS

The study included a total of 8 children with Noonan syndrome. All children were diagnosed at Jiangxi Provincial Children's Hospital between November 2017 and June 2021, which states that pathogenic variants or probable pathogenic variants are detected in children with the Noonan syndrome phenotype (Johnston et al., 2018). Among the eight affected children, there were four boys and four girls. The age at diagnosis ranged from 1 year and 7 months to 12 years and 6 months, with a median diagnosis age of 8 years and 7 months and 7 children presented with stunted growth.

To determine the cause of the condition, peripheral blood specimens were collected from the child and both parents with informed consent. The specimens were analyzed through exome second‐generation sequencing with pre‐documented and parental PCR‐NGS validation and evaluated according to the American College of Medical Genetics and Genomics (ACMG) guidelines for pathogenicity.

## 3 RESULTS

### 3.1 Clinical features

Apart from child 8's father, who was short with a height of 158 cm, all 7 children were diagnosed with stunted growth. Additionally, none of the parents showed any abnormalities in facial features or height. None of the parents were consanguineous and there was no family history of short stature‐related diseases. All the children had a birth weight of 2300–3500 g and a birth length of 48–50 cm, and their height at presentation was below −2SDS for the same age and sex. Four cases had structural cardiac abnormalities, including three cases of pulmonary stenosis, two cases of atrial septal defect (one of which had pulmonary stenosis combined with atrial septal defect) and one case of right aortic arch. In this study, GH cutoffs used <5 ng/mL for complete deficiency and 5–10 ng/mL for partial deficiency. All 7 children underwent growth hormone (arginine and clonidine) stimulation tests, with the exception of case 3, who was too young to undergo the tests. Four other cases indicated partial growth hormone deficiency, with three cases where peak growth hormone levels exceeded >10 ng/mL. Structural heart abnormalities were present in four children, and five children had varying degrees of intellectual‐motor retardation. All 8 children had unremarkable results in their chromosomal analysis (Table 1).

**TABLE 1:** Clinical characteristics of 8 children with Noonan syndrome.

Projects	Case1	Case 2	Case 3	Case 4	Case 5	Case 6	Case 7	Case8
Gender	Female	Male	Female	Female	Male	Female	Male	Male
Age	11 years and 8 months	6 years and 9 months	1 years and 7 months	10 years and 1 month	12 years and 6 months	12 years and 1 month	5 years and 11 months	8 years and 6 months
Birth weight (g)	3100	2300	2500	3300	3500	2800	3000	2950
Birth length (cm)	50	50	48	50	50	50	50	50
Height (cm)	121.8	93.0	66.8	121.0	129.5	123.0	99.0	111.2
Height (SDS)	−4.2	−5.7	−4.8	−3.0	−4.5	−4.6	−4.1	−4.0
Time of discovery of short stature	6 years	4 years	1 years	6 years	10 years	10 years	3 years	4 years
Annual elongation rate (cm)	4.0	3.1	4.8	4.0	3.5	3.4	4.6	3.2
GH excitation peak (ng/ml)	6.61	5.21	−	28.1	13.4	9.2	16.3	5.13
Chromosomes	46, XX	46, XY	46, XX	46, XX	46, XY	46, XX	46, XY	46, XY
Tanner staging	2 issues	1 issue	1 issue	2 issues	2 issues	2 issues	1 issue	1 issue
Characteristic signs	Droopy upper eyelids, low posterior hairline, shield‐like chest, wide breast spacing	Ear with small jaw	Wide eye spacing, small jaw, high jaw arch, low back hairline	Wide eye spacing, droopy eyelids, small jaw	Not obvious	Not obvious	Low ears, flat nose, eyes drooping upper eyelids, pectus carinatum	Eyes wide apart, neck short
Scoliosis	−	−	−	+	−	−	−	−
Echocardiography	−	−	Pulmonary artery stenosis, atrial septal defect	Pulmonary artery stenosis	−	Atrial septal defect	Right aortic arch	−
Abnormalities in intellectual development	−	+	+	+	−	+	+	−
Other	−	Osteochondroma of the right and left tibia discovered in December 19 after GH treatment	Traffic hydrocephalus with reduced white matter in the periventricular and semi‐oval	−	Caffeinated skin, multi‐pigmented nevus	Cutaneous strips of plush coffee spots	Surgery for incomplete testicular descent	Small testicular volume bilaterally and small measurement of both kidneys

### 3.2 Genetic examination

In this study, 8 children were utilized for the genetic examination research experiment. Our results showed that the *PTPN11* missense mutation was detected in 7 of the 8 children (Table 1), all of which were missense variants already reported by HGMD. The nucleotide and amino acid loci of the variants is shown in the Table 2, of which all were de novo variant, except for case 8, which was paternal (father had short stature of 158 cm, but no other abnormalities). In case 4, one heterozygous variant was detected in the *A2ML1*, c.436delG causing amino acid changes p. G146Afs*44 (frameshift variant), originating from the father (height 165 cm, no other abnormalities present).

**TABLE 2:** Genetic examination.

Case	Genotype	Nucleotide loci	Amino acid sites	Genes AND LOCI	Transcript numbering	Source	Pathogenicity analysis
1	*PTPN11*	c.844 A>G	p. I282V	*PTPN11*: chr12‐112910835	NM_002834	De novo	Pathogenic
2	*PTPN11*	c.1507G>A	p. G503R	*PTPN11*: chr12‐112926887	NM_002834	De novo	Pathogenic
3	*PTPN11*	c.854T>C	p. F285S	*PTPN11*: chr12‐112915455	NM_002834	De novo	Pathogenic
4	*A2ML1*	c.436delG	p. G146Afs*44	*A2ML1*: chr12‐9001390‐9001391	NM_0012 82424	Father	Likely pathogenic
5	*PTPN11*	c.836A>G	p. Y279C	*PTPN11*:chr12‐112910827	NM_0013 30437	De novo	Likely pathogenic
6	*PTPN11*	c.922A>G	p. N308D	*PTPN11*:chr12:112915523	NM_002834	De novo	Pathogenic
7	*PTPN11*	c.184T>G	p. Y62D	*PTPN11*:chr12:112888168	NM_002834	De novo	Pathogenic
8	*PTPN11*	c.794G>A	p. R265Q	*PTPN11*:chr12:112910785	NM_002834	Father	Likely pathogenic

Here, 7 children were treated with rhGH. One case did not undergo rhGH because patient 4 was 1 year and 7 months old and had pulmonary artery stenosis and atrial septal defect, communicating hydrocephalus, and decreased periventricular white matter. Parental concern was not focused on short stature, and parental consent for rhGH treatment was not obtained. The mean age at initiation of treatment was (9.6 ± 2.6) years, the mean birth length was 50.0 cm, the mean birth weight was (2992.9 ± 383.4) g. Five children were combined with growth hormone deficiency and one was completely deficient. The mean dose of rhGH treatment in the 7 children was (0.15 ± 0.05) IU/kg‐d and the duration of treatment ranged from 6 months to 2 years, with a mean of (1.4 ± 0.7) years. The growth rate increased after rhGH treatment compared with that before treatment [3.7 ± 0.5 cm/year before treatment, 8.0 ± 1.0 cm/year after treatment, *p* < 0.01], with the maximum growth rate between 3 and 6 months of treatment and decreasing thereafter as the treatment duration increased. The SDS of IGF‐1 is increased in all 7 children treated with rhGH, but remained within normal limits.

In summary, our result suggests that rhGH treatment improves the growth rate of children with Noonan syndrome short stature during treatment. 2 children had scoliosis with a cobb angle of less than 15 degrees before receiving growth hormone treatment, and the cobb angle did not increase on review of the spine film after treatment. 7 children treated with rhGH maintained normal blood glucose and thyroid function, and had no symptoms of headache, vision loss associated with increased intracranial pressure, joint enlargement and water‐electrolyte imbalance. One of the children was diagnosed with osteochondroma in both the right and left tibia six months after treatment, leading to the discontinued of growth hormone treatment. Furthermore, the study findings indicate that there were no significant negative impacts observed in children with Noonan syndrome who underwent rhGH treatment. Nevertheless, it is advisable to assess bone metabolism anomalies, especially in children with the *PTPN11* pathogenic variant, before administering the medication as well as to monitor scoliosis and osteochondroma during their post‐treatment phase.

## 4 DISCUSSION

Noonan syndrome (OMIM 163950) was first reported by Noonan in 1968 (Noonan, 1968) and is characterized by a peculiar facial appearance, pulmonary artery stenosis, short stature, growth retardation and coagulation disorders (Tafazoli et al., 2017). The disease can occur in both sexes, is autosomal dominant, and can be disseminated or run‐in families. Recent advances in molecular biodiagnostics have led to a more comprehensive understanding of the causes (Johnston et al., 2018). Growth retardation is seen in almost all children with Noonan syndrome, and all 8 children in this group met the diagnostic criteria for short stature. Noonan Syndrome can be diagnosed by observing the characteristic facial features that appear during infancy and early childhood. These features include a large forehead, wide‐spaced eyes, drooping upper eyelids, a low nasal bridge with a full nasal tip, a small jaw, a short neck or webbed neck, pronounced facial wrinkles, a pronounced nasolabial fold, and low ear position. However, as the child grows older, these facial features may become less distinct. As individuals with Noonan syndrome age, their facial features may become increasingly atypical. However, according to our study, in the cases of adolescent males with no specific facial features, the only exception being case 8 whose father had a height of 158 cm, their parents had no abnormal height or specific facial features. It is worth noting that there was no familial link between any of the cases presented here.

Short stature is a primary symptom of Noonan syndrome and is the reason many children with the condition are diagnosed (Yart & Edouard, 2018). Effective treatment of short stature in children with Noonan syndrome is essential to ensure they have a satisfying life's quality. Data from short‐term and long‐term studies have shown that rhGH treatment can increase the growth rate and improve height in children with Noonan syndrome (Gkourogianni et al., 2016; Wu et al., 2022). Bamba and Kanakatti Shankar (2022) conducted a controlled study of rhGH treatment in children with Noonan syndrome. Horikawa et al. (2020) evaluated the efficacy of rhGH in children with Noonan syndrome short stature in a multicenter, randomized, double‐blind, multi‐dose (low and high) parallel controlled trial, showing a significant height benefit in the high‐dose group after 4 years of treatment. In children, the dose of GH therapy should be based on the percentile of height. Typically, the recommended dose for Noonan syndrome is 0.066 mg/m^2^.d. Our study shown that the earlier rhGH treatment is started, the better the outcome. In addition, because the incidence of leukemia and some solid tumors in patients with Noonan syndrome is higher than that in the general population (Noonan & Kappelgaard, 2014), it is recommended that close observation and follow‐up of tumor susceptibility should be performed when rhGH therapy is initiated. Moreover, dose adjustments based on growth rate, body mass change, and IGF‐1 levels are required. The level of IGF‐1 should be maintained within the normal range during the treatment. In the case of good adherence, if growth is unsatisfactory and IGF‐1 levels are low, rhGH doses may be increased within the approved dose range. Dose reduction may be considered after the first 2 years if serum IGF‐1 levels are above the normal range, especially if they persist above 2.5 SDS. In this study, serum IGF‐1 levels in 8 children with Noonan syndrome were in the normal range after rhGH treatment, although they were higher than before treatment. Additionally, the average duration of rhGH treatment in the 8 children with Noonan syndrome was (1.2 ± 0.9) years, with a significant increase in growth rate during the treatment period, with the greatest growth rate occurring between 3 and 6 months of treatment and decreasing thereafter as the treatment period progressed.

The efficacy and safety of rhGH therapy in patients with Noonan syndrome cannot be ignored. It is important to note that children with Noonan syndrome are at potential risk for tumors and hypertrophic cardiomyopathy, and given the mitogenic effects of rhGH, cardiac function and tumor risk should be monitored closely during treatment (Noonan & Kappelgaard, 2014). No serious adverse effects have been reported in studies of rhGH in Noonan syndrome (Rodríguez et al., 2021). rhGH treatment did not affect BMI, it resulted in favorable changes in fat mass and body composition. Growth hormone therapy may be associated with metabolic abnormalities, heart disease, and tumor‐related disorders (Noonan & Kappelgaard, 2014). Moreover, rhGH treatment promotes linear growth in prepubertal children with Noonan syndrome, and rhGH treatment is well tolerated in patients with Noanan syndrome, including in patients with previous cardiovascular diseases comorbidities and those receiving concomitant medications all cardiovascular diseases complications were reported as comorbidities (Sodero et al., 2023).

Noonan syndrome is characterized by a variety of symptom, with pulmonary stenosis being the most common manifestation. Valvular dysplasia and myocardial hypertrophy are also frequently observed, with an incidence of approximately 50% to 60% (Yart & Edouard, 2018). In our study, four of the children had structural heart abnormalities that included pulmonary stenosis, atrial septal defect, and right aortic arch. The etiology of Noonan syndrome is related to genes variants associated with the cellular Ras/MAPK pathway, leading to increased signaling in the pathway. To date, *PTPN11* is the most common related genes have been reported to be associated with Noonan syndrome, which contains 15 exons and 14 introns with a full gene length of approximately 91,182 bp, and is an important signaling molecule for the cell. 50% of Noonan syndrome is associated with missense variants in *PTPN11*, resulting in abnormalities in both the N‐SH2 and PTP functional domains are abnormal (Grant et al., 2018). Mutations are concentrated in Exon3 (N‐SH2 functional domain), Exon7, Exon8 and Exon13 (PTP functional domain) (Jongmans et al., 2005). The protein product encoded by *PTPN11*, SHP‐2, plays a key role in the embryonic development of heart valves, which is consistent with the high prevalence of valve lesions in Noonan syndrome. SHP‐2 is also involved in signaling processes such as growth hormone, growth factors and fibroblast factors, leading to short stature and skeletal abnormalities in children. Seven of the eight children in this group also had missense variants in *PTPN11*, all of which were considered pathogenic according to the ACMG guidelines (Amendola et al., 2016). In addition to the *PTPN11* mutations in the RAS‐MAPK signaling pathway, including *KRAS*, *SOS1*, *RAF1*, *SHOC2*, *NRAS*, *BRAF* and *CBL*, can also cause Noonan syndrome (Grant et al., 2018). The *PTPN11* mutation causes the most severe short stature in children, which may also be one of the reasons why children are detected early.

Currently, there is limited information available on Noonan syndrome in China, and more clinical research is needed to better understand its clinical and molecular diagnostic aspects. Two of the children in our study had scoliosis with a cobb angle of less than 15 degrees before receiving growth hormone therapy. However, after reviewing the spine film post‐treatment, the cobb angle did not increase. One child, who was found to have osteochondroma of the right and left tibia six months after treatment, had growth hormone therapy discontinued, and regular follow‐up was recommended due to the *PTPN11* mutation (Tajan et al., 2018). Therefore, it is recommended to pay attention to bone metabolism abnormalities, especially in children with *PTPN11* mutation before starting treatment. Moreover, close surveillance for scoliosis and osteochondroma during the follow‐up process is essential.

## AUTHOR CONTRIBUTIONS

Xian Wu, Lirong Yu and Jiali Wu designed the overall research strategy. Li Yang collected the clinical information. Xian Wu wrote the manuscript. Xian Wu, Jiali Wu, Yi Yuan, and Lirong Yu participated in data discussion. All authors contributed to the article and approved the submitted version.

## FUNDING INFORMATION

This work was supported by grants from the General Health Plan of Jiangxi Province (202130868).

## CONFLICT OF INTEREST STATEMENT

The authors declare that the research was conducted in the absence of any commercial or financial relationships that could be construed as a potential conflict of interest.

## ETHICS APPROVAL AND CONSENT TO PARTICIPATE

The authors are accountable for all aspects of the work in ensuring that questions related to the accuracy or integrity of any part of the work are appropriately investigated and resolved. The study was conducted in accordance with the Declaration of Helsinki (as revised in 2013). The study was approved by Institutional Review Board of Jiangxi Provincial Children's Hospital (JXSETYY‐YXKY–20220131). All the study subjects provided informed consent.

## Data Availability

No data availability.
